# A comparison of weather variables linked to infectious disease patterns using laboratory addresses and patient residence addresses

**DOI:** 10.1186/s12879-018-3106-9

**Published:** 2018-04-27

**Authors:** Abdelmajid Djennad, Giovanni Lo Iacono, Christophe Sarran, Lora E. Fleming, Anthony Kessel, Andy Haines, Gordon L. Nichols

**Affiliations:** 1 Public Health England, London, UK; 2 School of Veterinary Medicine, University of Surrey, Guildford, UK; 3 Centre for Radiation Chemical and Environmental Hazards, Public Health England, Harwell, Didcot, UK; 4 Met Office, Exeter, UK; 5 European Centre for Environment and Human Health, University of Exeter, Exeter, UK; 6 London School of Hygiene and Tropical Medicine, London, UK; 7 University of East Anglia, Norwich, UK; 8 Statistics, Modelling and Economics Department, National Infection Service, Public Health England, 61, Colindale Avenue, London, NW9 5EQ UK

**Keywords:** *Campylobacter*, *Cryptosporidium*, Temperature, Rainfall, Data linkage, SGSS, MEDMI, MIDAS

## Abstract

**Background:**

To understand the impact of weather on infectious diseases, information on weather parameters at patient locations is needed, but this is not always accessible due to confidentiality or data availability. Weather parameters at nearby locations are often used as a proxy, but the accuracy of this practice is not known.

**Methods:**

Daily *Campylobacter* and *Cryptosporidium* cases across England and Wales were linked to local temperature and rainfall at the residence postcodes of the patients and at the corresponding postcodes of the laboratory where the patient’s specimen was tested. The paired values of daily rainfall and temperature for the laboratory versus residence postcodes were interpolated from weather station data, and the results were analysed for agreement using linear regression. We also assessed potential dependency of the findings on the relative geographic distance between the patient’s residence and the laboratory.

**Results:**

There was significant and strong agreement between the daily values of rainfall and temperature at diagnostic laboratories with the values at the patient residence postcodes for samples containing the pathogens *Campylobacter* or *Cryptosporidium*. For rainfall, the R-squared was 0.96 for the former and 0.97 for the latter, and for maximum daily temperature, the R-squared was 0.99 for both. The overall mean distance between the patient residence and the laboratory was 11.9 km; however, the distribution of these distances exhibited a heavy tail, with some rare situations where the distance between the patient residence and the laboratory was larger than 500 km. These large distances impact the distributions of the weather variable discrepancies (i.e. the differences between weather parameters estimated at patient residence postcodes and those at laboratory postcodes), with discrepancies up to ±10 °C for the minimum and maximum temperature and 20 mm for rainfall. Nevertheless, the distributions of discrepancies (estimated separately for minimum and maximum temperature and rainfall), based on the cases where the distance between the patient residence and the laboratory was within 20 km, still exhibited tails somewhat longer than the corresponding exponential fits suggesting modest small scale variations in temperature and rainfall.

**Conclusion:**

The findings confirm that, for the purposes of studying the relationships between meteorological variables and infectious diseases using data based on laboratory postcodes, the weather results are sufficiently similar to justify the use of laboratory postcode as a surrogate for domestic postcode. Exclusion of the small percentage of cases where there is a large distance between the residence and the laboratory could increase the precision of estimates, but there are generally strong associations between daily weather parameters at residence and laboratory.

## Background

Weather can contribute significantly to the occurrence of many infectious diseases, and high spatial resolution of exposure data has increasingly been recognized as a requirement in quantifying these relationships [1]. These analyses can help in understanding the likely changes in infectious diseases that may take place in the future through climate or other environmental change [2, 3], particularly in determining the drivers for change [4, 5], in the application of suitable models, and for designing interventions [6]. Variations in diseases by country have been used to examine the impact of weather that is separated from geography [7, 8]. For example, environmental weather parameters at low resolution (e.g. national average) have been used as a proxy for the weather experienced by an individual. The methods of collection for a range of different weather parameters differ, often based on comparatively few weather stations with limited interpolation of the data at other locations or because the exact location of the exposed individual is not known.

To understand the impacts of weather (and the general environment) on both communicable and non-communicable diseases, it is therefore desirable to have information on the weather parameters as close as possible to the location of people’s residence. This is not always possible for a variety of reasons. Datasets from different agencies are seldom linked together, although important efforts to integrate heterogeneous datasets have been made (see the MEDMI project at https://www.data-mashup.org.uk). With the movement of reported geographic data from the local to the national to the international infectious disease surveillance records [9], the geographic resolution can be ‘lost’ in this process. For example, records from diagnostic microbiology laboratories for England and Wales in the Public Health England Second Generation Surveillance System (SGSS) database have had limited patient postcode data before 2008. Furthermore, the computational resources needed for data linkage at the individual patient postcode level can be significant if large infectious disease datasets are being interrogated. As an example, a study found over a million *Campylobacter* infections recorded in England and Wales [10]. The linkage with patient postcodes also presents challenges to comply with ethical and legal requirements of confidentiality [11], with insufficient evidence about the efficacy of some anonymisation methods [12].

These concerns are particularly relevant for the increasing number of initiatives aiming to integrate data from different sources (e.g. big data mashups), particularly the linkage of human health data with environmental data needed to study the effects of climate and other environmental change on human diseases [13–15]. This study sought to test whether the linkage of weather parameters in England and Wales to the diagnostic laboratory postcodes for daily *Campylobacter* and *Cryptosporidium* cases across England and Wales reported to Public Health England would be suitable for use as a proxy for the weather at the patient’s residence postcode. The linkage of interpolated local weather parameters supplied by the UK Met Office with the postcodes of the diagnostic laboratories instead of with patient residence postcodes would address many of the concerns above.

## Methods

### Infectious diseases data

The Second Generation Surveillance System (SGSS) database of Public Health England was used to extract daily individual records of *Campylobacter* and *Cryptosporidium* infections from 01/01/2005 to 31/12/2014 using specimen date, domestic residence postcodes and laboratory postcodes. There are approximately 11,200 postcode sectors in the UK, each containing approximately 3000 addresses, the size of the postcode sectors varies, ranging from 2864 km^2^ in a low populated region in the Scottish Highlands to approximately 0.001 km^2^ in most of the densely populated sectors of London (according to BPH Postcodes at https://www.bph-postcodes.co.uk/guidetopc.cgi). The two pathogens were chosen because they were thought to be transmitted by different routes (*Campylobacter* from chicken and *Cryptosporidium* from faecal contamination of water); and have a different seasonality (*Campylobacter* peaks in late spring and *Cryptosporidium* in late summer) and may therefore be subject to different weather influences (although the postcode relationships to weather were not thought to be influenced by these). *Cryptosporidium* infections commonly occur as local outbreaks as the transmission is waterborne [16]; both outbreaks and sporadic infections may be more influenced by rainfall. *Campylobacter* is seasonal and sporadic, and more linked to a mixture of seasonal drivers [10]. There were more *Campylobacter* cases than *Cryptosporidium* cases, and both had country-wide distributions.

### Meteorological data

The meteorological data used in this analysis were held on the Medical and Environmental Data a Mash-up Infrastructure (MEDMI) platform [13] (see http://www.data-mashup.org.uk). The MEDMI database includes the Met Office weather observations datasets from the Met Office Integrated Data Archive System (MIDAS). The MIDAS datasets consist of fixed station observations and derived climate statistics; these include over 1500 sites measuring temperature and over 12,000 sites measuring rainfall.

### Data linkage

The laboratory postcode, patient residence postcode, and patient specimen date of *Campylobacter* and *Cryptosporidium* infections were linked with daily temperatures (minimum and maximum ^o^C) and daily rainfall (mm) using the MEDMI Database tools. The Public Health England ethics committee approval and safeguards of patient data were in place to ensure no breach of patient confidentiality.

Estimates of temperature and rainfall were obtained for the centroid of each laboratory and patient residence postcode by Inverse-Distance-Weighted (IDW) interpolation [17]. The estimates were calculated as the inverse-distance-weighted arithmetic mean of temperature and rainfall measurements taken at weather observation sites within 50 km of each centroid.

The spatial-temporal linkage of infectious diseases cases with meteorological data on the MEDMI platform represents an important realisation of Geographical Information System (GIS) that can be used by scientists and policy makers to generate and test hypotheses, to facilitate the detection of potential associations between weather data and infection occurrence, and to estimate the risk of infection [18].

### Statistical analysis

The daily measurements of rainfall (mm), maximum and minimum temperatures (°C) at the residence postcodes were plotted against the daily measurements of rainfall, maximum and minimum temperatures at the laboratory postcodes. A linear regression in R statistical software [19] was used to examine associations between the weather variables at domestic and laboratory postcodes, where the daily rainfall or temperature at the residence postcodes was the response variable, and the daily rainfall or temperature at laboratory postcodes was the explanatory variable for each pathogen (*Campylobacter* and *Cryptosporidium*). In addition, we assessed the potential dependence of the findings on the absolute and relative locations of patients and laboratories by using R statistical software and GIS.

The distance between patient and laboratory (here and throughout *patient-laboratory distance*) was measured as the great-circle distance (i.e. the shortest distance between two points on the surface of a sphere), calculated by using the *haversine* formula [20] between the latitude and longitude of patient location and the corresponding laboratory where the specimen was sent for diagnosis. For each laboratory, we also calculated the mean distance of the patient residence from the diagnostic laboratory; and we refer to this as the *local mean patient-laboratory distance*, where *local* emphasizes that the patient-laboratory distances were averaged over all cases within the catchment area of that particular laboratory. Similarly, for each laboratory, we calculated the means and standard deviations of the differences in daily rainfall, minimum and maximum temperature between the patient residence address and the corresponding laboratory locations.

## Results

There were 441,203 *Campylobacter* infections, of which 11,381 had missing residence postcode information. Records with missing residence postcodes were removed from the study dataset, leaving 429,822 cases with both residence and laboratory postcodes. For *Cryptosporidium* infections, there were 30,970 cases with 785 missing residence postcode information, leaving 30,185 cases with both residence and laboratory postcodes.

**Fig. 1:**
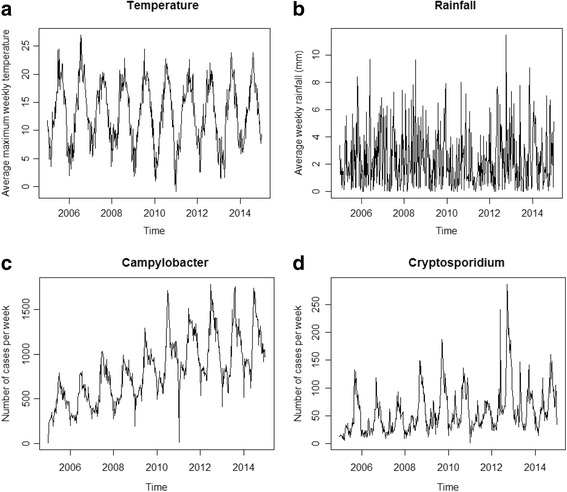
Time series of maximum temperature, average rainfall, *Campylobacter* and *Cryptosporidium* cases in England and Wales. Panels **a**, **b**, **c** and **d** show the average maximum temperature (°C) per week at laboratory postcodes, the average total rainfall (mm) per week at laboratory postcodes, the number of *Campylobacter* and *Cryptosporidium* cases per week from 01/01/2005 to 31/12/2014 in England and Wales

The time series of average maximum weekly temperature and average weekly rainfall at laboratory postcodes are shown in Fig. 1 (a and b). The weekly number of cases of *Campylobacter* and *Cryptosporidium* are shown in Fig. 1 (c and d).

**Fig. 2:**
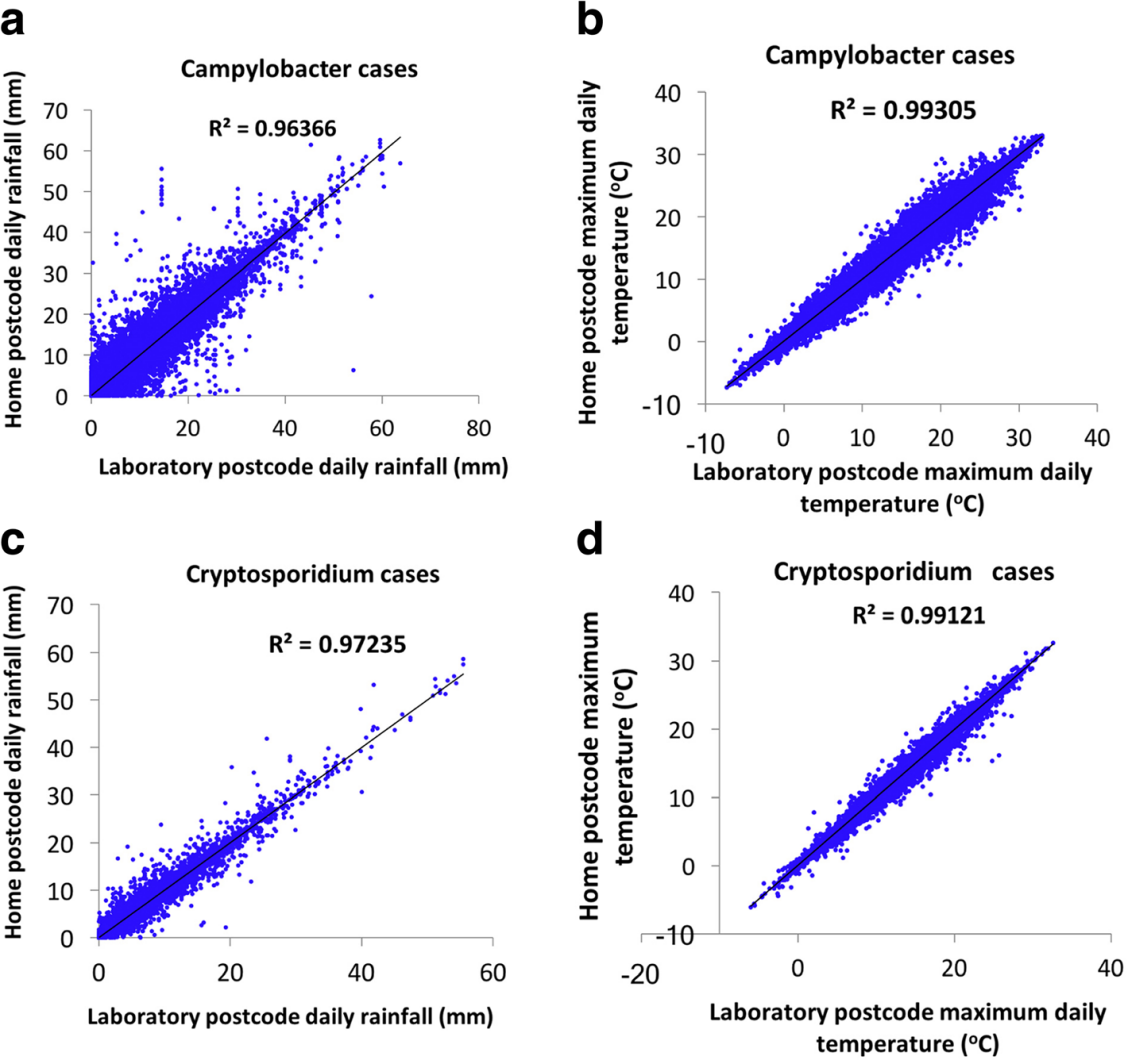
Association between patient residence and laboratory measurements. Panels **a**, **b**, **c** and **d** show the association between the maximum daily temperature (°C) and daily rainfall (mm) which were measured at patients residence postcodes and laboratory postcodes from *Campylobacter* and *Cryptosporidium* daily infections of these patients

Figure 2 (panels a, b, c and d) shows strong associations between the daily measurements of rainfall (mm) and temperature (°C) obtained at residence postcodes and the daily measurements of rainfall (mm) and temperature (°C) obtained at laboratory postcodes for both pathogens (*Campylobacter* and *Cryptosporidium*). Because both temperature variables gave similar results for *Campylobacter* and *Cryptosporidium* cases, we plotted the figures for the maximum temperature.

For maximum and minimum daily temperatures at the laboratory postcodes, the estimates of the slope coefficients was 0.99 (*p*-value ≤0.001) and R-squared was 0.99 for both pathogens. For daily rainfall at the laboratory postcodes, the estimates of the slope coefficients was 0.99 (p-value ≤0.001) and R-squared was 0.96 for *Campylobacter* and 0.99 (p-value ≤0.001) and R-squared was 0.97 for *Cryptosporidium* as shown in Fig. 2.

**Fig. 3:**
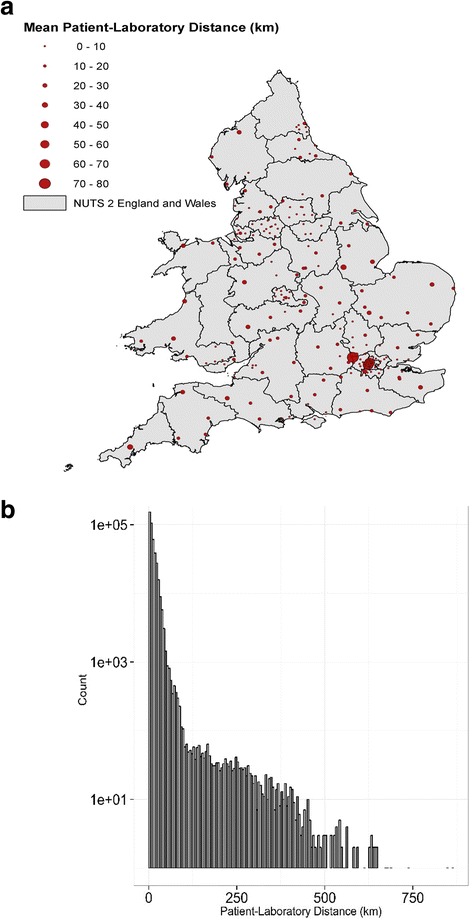
Distribution of patient-laboratory distances. Panel **a**: geographic location of diagnostic laboratories in England and Wales. The size of the bubble is proportional to the local mean patient-laboratory distance. For visual purposes, the territory is divided according to NUTS 2 classification. Panel **b**: Statistical distribution of patient-laboratory distances

The distribution of the distances between patient residence address and the corresponding laboratory address is shown in Fig. 3. Although the overall (i.e. averaged over the entire dataset) mean patient-laboratory distance is only 11.9 km (standard deviation = 19.8 km), the distribution exhibits a heavy tail with some rare situations where the patient-laboratory distance was larger than 500 km. This was confirmed by a detailed inspection of the data; for example, there were few instances where the diagnostic laboratory was located in South England and the patient residence in Northern Ireland or Scotland.

**Fig. 4:**
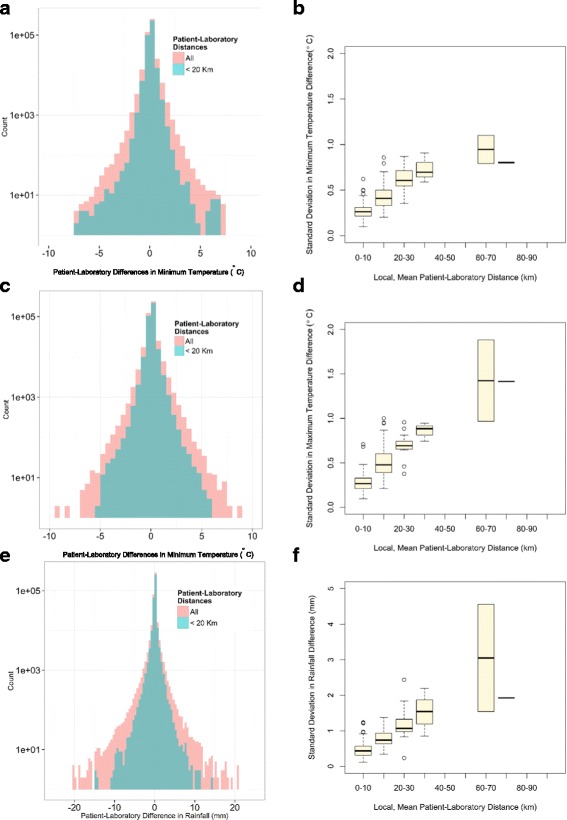
Impact of patient-laboratory distances. Panels **a**, **c** and **e**: Histogram showing the distributions of the differences in minimum temperature, maximum temperature and rainfall registered at the laboratory and patient residence addresses. The blue histograms correspond to the subset of cases when the patient-laboratory distances are lower than 20 km. Panels **b**, **d** and **f**: the x-axis represents the local mean patient-laboratory distances (for visual purposes the continuous values were converted into a discrete bins); the y-axis is standard deviation in minimum temperature, maximum temperature and rainfall differences registered at these addresses

Figure 4 (a, c and e) shows the distributions of the differences in minimum temperature, maximum temperature and rainfall registered at the laboratory and patient addresses. The distributions exhibited a tail heavier than the corresponding exponential fit, with differences up to ±10 °C for the minimum and maximum temperature and ± 20 mm for rainfall. Exponential fits are commonly used as a benchmark for heavy tailed distributions, although other approaches have been proposed [21].

An important property of heavy tailed distributions is that they result in a higher probability of observing the values with many outliers. To test if the heavy tailed distributions arose from the exceptional instances of large patient-laboratory distances, we compared the distributions based on the entire dataset (red histograms in Fig. 4a, c, e), with the distribution based on the subset of cases when the patient-laboratory distances are lower than 20 km (blue histograms). The comparison confirmed that large discrepancies in the temperatures and rainfall between laboratory and patient residence tended to occur for only the large patient-laboratory distances.

Figure 4 (panels b, d and f) shows the standard deviation in rainfall, minimum and maximum temperatures difference versus the local mean patient-laboratory distances. As expected, when this distance was larger (e.g. because of a large laboratory catchment area), then the difference in the climatic variables between patient residence and laboratory were more scattered, resulting in a larger standard deviation.

Note that the weather stations used to link both laboratory and residence postcodes in the analysis were likely to be the same, but the interpolations used different weightings according to location and thus predominantly gave different results.

## Discussion

There is a growing need for a more ecological view of public health [22], and the associations linking data on weather with disease occurrence can be extremely useful in demonstrating the public health burden associated with climate change [23]. It is becoming evident that with many infectious diseases, the impacts of weather on seasonality can be teased out through the linkage of local weather parameters to cases [24]. There is a good rationale for developing methods to easily link disease data to weather and other environmental drivers, and to provide the tools to achieve this. For infectious diseases, linkage can be examined across pathogens; and this is being developed through the extraction of long-term data on individual local weather parameters that can easily be linked through the postcode of the laboratory where the case was diagnosed. In this study, the differences between the weather variables associated with the patient residence postcode and the laboratory postcode were comparatively small and were mostly as a result of a small number of cases whose domestic residence was at a great distance from the laboratory.

The use of the Inverse-Distance Weighted (IDW) interpolation tended to smooth the values spatially leading to a higher correlation between laboratory and residence locations (especially in urban areas where the two locations are likely to be close); more sophisticated methods would require altitude data and additional geo-processing. Gridded datasets that use the IDW method with statistical adjustments for known atmospheric effects have been used by some researchers [25, 26], but unadjusted IDW interpolation provides a more spatially localised estimate than taking a single value for a region or a country (e.g. the Central England Temperature) used by [27] or [28]), or simply by taking the measurements from nearby weather stations [29].

The analysis is straightforward and applicable to situations resembling the same circumstances in England and Wales, i.e. regions with same climatic zones such as regions in the same Koppen-Geiger classification [30], and with similar public health capacity and spatial density of diagnostic laboratories. The study can be relevant to other situations; for example, the findings are expected to be valid in countries in climatic zones exhibiting smaller spatial variance in temperature and rainfall and/or shorter patient-laboratory distances. However, it is possible that the relationship we found between weather parameters at the laboratory and residence postcodes may not transfer to other regions and countries where the health service administrative boundaries are on different scales and data distribution is different. In these cases, an assessment like the present study would be needed.

The analysis was done only for rainfall and temperature; however, some diseases may be affected by other environmental variables (not only climatic such as humidity and barometric pressure but also soil type, density of livestock etc.). Our analysis was based on outdoor weather variables and was not able to assess the seasonal indoor environmental variables that most people are exposed to. The underlying assumption was that most people spend longer time at home, however, it is estimated that people spend up to 40% of time at locations other than their residence address (schools, workplace, transport).

## Conclusion

Using the weather parameters at laboratory postcode as a proxy for the weather parameters at the patient residence can be a valid approach in England and Wales and useful in exploring the relationships between weather and infectious diseases over time. However, large discrepancies between the weather parameters at the two different locations can occur in some cases. These typically arise in instances of large distances between patient residence address and the diagnostic laboratory, but also reflect the intrinsic spatial variability of both temperature and rainfall (e.g. localized episodes of rainfall). Data linkage at laboratory postcode level also ensures data confidentiality.

## Abbreviations

GIS: Geographical Information System
IDW: Inverse Distance Weighted
MEDMI: Medical & Environmental Data Mash-up Infrastructure
MIDAS: Integrated Data Archive System
SGSS: Second Generation Surveillance System
